# Cryopreservation Causes Genetic and Epigenetic Changes in Zebrafish Genital Ridges

**DOI:** 10.1371/journal.pone.0067614

**Published:** 2013-06-21

**Authors:** Marta F. Riesco, Vanesa Robles

**Affiliations:** INDEGSAL and Molecular Biology, University of León, León, Spain; Temasek Life Sciences Laboratory, Singapore

## Abstract

Cryopreservation is an important tool routinely employed in Assisted Reproduction Technologies (ARTs) and germplasm banking. For several years, the assessment of global DNA fragmentation seemed to be enough to ensure the integrity of genetic material. However, cryopreservation can produce molecular alterations in key genes and transcripts undetectable by traditional assays, such modifications could interfere with normal embryo development. We used zebrafish as a model to study the effect of cryopreservation on key transcripts and genes. We employed an optimized cryopreservation protocol for genital ridges (GRs) containing primordial germ cells (PGCs) considered one of the best cell sources for gene banking. Our results indicated that cryopreservation produced a decrease in most of the zebrafish studied transcripts (*cxcr4b, pou5f1, vasa* and *sox2*) and upregulation of heat shock proteins (*hsp70, hsp90*). The observed downregulation could not always be explained by promoter hypermethylation (only the *vasa* promoter underwent clear hypermethylation). To corroborate this, we used human spermatozoa (transcriptionally inactive cells) obtaining a reduction in some transcripts (*eIF2S1*, and *LHCGR*). Our results also demonstrated that this effect was caused by freezing/thawing rather than exposure to cryoprotectants (CPAs). Finally, we employed real-time PCR (qPCR) technology to quantify the number of lesions produced by cryopreservation in the studied zebrafish genes, observing very different vulnerability to damage among them. All these data suggest that molecular alterations caused by cryopreservation should be studied in detail in order to ensure the total safety of the technique.

## Introduction

Cryopreservation is a technique widely used in Assisted Reproduction Technologies (ARTs) as well as in germplasm banking. Although this technology has been used for several years, recent studies have pointed out that it can cause molecular alterations in cells at different levels [1] that may interfere with normal embryo development. For several years, the assessment of global DNA integrity (by fragmentation assays) seemed to be enough to ensure the efficiency of cryopreservation in preserving genetic material [2,3]. However, a number of other types of damage are known to occur. Deletions, creation of abasic sites, base modifications or DNA crosslinks are different types of DNA damage [4] that cannot be detected by the above-mentioned methods. It is well documented that spermatozoa DNA damage can lead to poor fertilization, but more importantly, it can also impair embryonic development, increase the number of abortions or even produce childhood diseases [5]. But DNA is not the only crucial factor to be analyzed at molecular level. It is a fact that cryopreserving human sperm decreases or even eliminates the presence of certain transcripts [6]. The importance of spermatozoa mRNAs has only recently been revealed. Recent studies have proposed that some spermatozoa transcripts are crucial for fertilization and even for early embryo development [7]. The potential association of ARTs with a higher incidence in human imprinting disorders, such as Angelman and Beckwith-Wiedemann syndromes [8], has also been suggested. However, to date only a few studies in mouse and human oocytes have investigated cryogenic effects on imprinted methylation [9–11]. The relationship between promoter DNA methylation and gene expression is well described in the literature [12] and any modification potentially produced by cryopreservation in the normal promoter methylation pattern in crucial genes deserves special attention.

All these data suggest that the study of molecular modifications potentially produced by cryopreservation on key transcripts could be relevant not only in ARTs in humans but also in any species suitable for cryobanking. To study these questions, we used zebrafish as a model. Zebrafish has become an important animal model for biomedical research and several transgenic and mutant lines are being created and should be cryopreserved. More importantly, this species is an ideal model for investigating cancer [13,14] and other human diseases, different infectious [15], neurodegenerative disorders [16], and vascular disorders [17]. Several studies have shown a close relationship between zebrafish and the human genome [18–20], and remarkable conservation between human and zebrafish genes has been shown [21]. Moreover, many similar tumor genes and pluripotency factors; nanog homeobox (*nanog*), POU domain, class 5, transcription factor 1 (*oct4/pou5f1*), SRY-box containing gene 2 (*sox2*) and Krüppel-like factor 4b (*klf4*), have a conserved expression pattern in zebrafish and humans [22,23] and similar methylation patterns have been also obtained [24].

The aim of the present study was to determine whether an optimized cryopreservation protocol (in terms of cell survival and DNA fragmentation) produces alterations at genetic and epigenetic levels using a candidate gene approach, since different genome regions are known to have different susceptibility to damage. We carried out this study in zebrafish genital ridges (GRs), which contain primordial germ cells (PGCs), and are suggested by many authors to be the best cellular source for cryobanking in this species, since both paternal and maternal genomes can be preserved [25]. The cryopreservation protocol, developed by our group, was optimal for these cells taking into account viability, DNA integrity and cell functionality [26]. Our work focused on genes and transcripts with important roles in pluripotency and PGC viability. We selected genes expressed in PGCs: Dead end (*dnd*), crucial to PGC survival and migration [27], vasa (*vasa*) relevant in germ cell lineage and PGC development [28], chemokine (C-X-C motif) receptor 4b (*cxcr4b*) involved in PGCs migration [29]; genes with important functions in stem cells pluripotency: POU domain, class 5, transcription factor 1 (*pou5f1*) [30]; and genes involved in differentiation processes in early embryo development: SRY-box containing gene 2 [31] and 3 [32] (*sox2* and *3*) (Supplementary Table 1 (Figure S1)).

The effect of cryopreservation on gene expression and promoter methylation was studied. Also, the number of lesions after cryopreservation was quantified in those key genes by real-time PCR (qPCR). This technique is based on blocking thermostable DNA polymerase progression by lesions in the DNA template which results in a decrease in DNA polymerase fidelity and amplification efficiency [33].

This study represents a step forward in the knowledge of the effects of cryopreservation at molecular level, questioning whether more in-depth molecular studies would be advisable to guarantee the total safety of the technique.

## Material and Methods

### Ethics statement

The experiments carried out in this study using embryos from zebrafish are part of a project from the Ministerio de Ciencia e Innovación AGL2009-06994 specifically approved by the University of León Bioethical Committee (https://www.unileon.es/investigadores/comite-etica). The University of León is authorized by the Ministry of Agriculture, Fisheries and Food to breed zebrafish. The use of human spermatozoa for RNA extraction and the Written Informed Consent (IC) were approved by the University of León Bioethical Committee as part of another grant application (ERC starting grant). ICs were obtained from donors.

### Zebrafish maintenance and genital ridge dissection

Adult zebrafish (*Danio rerio*) vasa EGFP zf45 strain, tg{vas: egfp} transgenic line, generated by the Olsen lab [34], were maintained in tanks under standard conditions [35] and were used exclusively for embryo production. For embryo collection, adult zebrafish were kept in glass tanks at 28 ^°^C with 6 fish per tank at the ratio of 1:2 male: female. The GRs were manually excised from 26-somite embryos using fine watchmaker’s forceps under a microscope (Nikon, Japan), as described by Kobayashi [36]. PGCs obtained at later stages could decrease their migration ability [37]. The GRs were kept in modified Leibovitz medium (L15) supplemented with 5% fetal bovine serum (FBS) at room temperature (RT) until further use.

### Genital ridge exposure and cryopreservation

In order to study the effect of cryoprotectants and cryopreservation on gene expression, promoter methylation and DNA damage in PGCs and the surrounding somatic cells, genital ridges were exposed to cryoprotectants; 5 M dimethyl sulfoxide (DMSO), 1 M ethylene glycol (EG), 4% polyvinyl-pyrrolidone (PVP), in a three step-wise manner. First, they were placed in a pretreatment solution with 2 M DMSO and 0.5 M EG in Hank’s premix (138.28 mM NaCl, 5.42 mM KCl, 0.255 mM Na_2_HPO_4_, 0.455 mM KH_2_PO_4_, 1.3 mM CaCl_2_, 1.0 mM MgSO_4_) for 10 min. The samples were then exposed to 5 M DMSO and 1 M EG in Hank’s premix for 2 m. In the last step, external cryoprotectant agent (PVP) was added for 2 min. The GRs containing PGCs, were loaded into 0.5 mL straws and exposed to liquid nitrogen vapor (2 cm over the surface) for 20 min and plunged into liquid nitrogen [26].

### Human sperm sample cryopreservation

To know the effect of cryopreservation on mRNAs in transcriptionally inactive cells, human sperm samples were cryopreserved as follows: samples were diluted 1:1 in a commercial cryoprotective medium, Sperm Freezing Medium (Irvin, Spain), to a final concentration of 5 million/mL. The mixture was equilibrated for 10 min at RT and loaded into 0.5 mL French straws. Then, the straws were exposed to liquid nitrogen vapors (2 cm over the surface) for 30 min, plunged into liquid nitrogen and stored until used. Thawing was carried out at RT for 5 min. Cell morphology and motility were checked under light microscopy after cryopreservation.

### DNA damage assay

#### Hydrogen peroxide treatment

Hydrogen peroxide treatment was carried out as a positive control for DNA damage. Zebrafish GRs containing the PGCs for DNA extraction were exposed to hydrogen peroxide (H_2_O_2_) 3% (Merck, Spain) in L15 for 30 min at RT, and the H_2_O_2_ was washed twice with L15.

#### DNA isolation and purification

DNA was extracted from zebrafish GRs containing PGCs in fresh, cryoprotectant agent (CPAs) exposed, cryopreserved and H_2_O_2_ treated samples. The DNA Blood and Tissue Kit (Qiagen, Spain) was used following the manufacturer’s instructions. Approximately 75 GRs were used per extraction. DNA quantity and purity were determined using a nanodrop spectrometer (nanodrop 1000, Thermo Scientific, Biocompare, Spain). The isolated DNA showed high purity (A_260/280_ > 1.8) and was stored at -20^°^C until required for analysis.

#### Real time qPCR

The primers for qPCR were designed using Primer Express (Software v2.0, Applied Biosystems) and Primer Select (Software v 10.1 DNA Star, Lasergene Core Suit). The amplicon was always over 600 bp. For internal normalization control, a pair of primers (forward and reverse) was designed within each studied amplicon. The primer nucleotide sequences and size of the amplicon can be seen in the supplementary material (Table S2).

The qPCR conditions were optimized for the different primers to achieve similar amplification efficiencies to compare different amplicons. The amplification was monitored and analyzed by the intercalation of the fluorescent dye, SYBR Green, to double-stranded DNA. Product specificity was tested by melting curves and product size was visualized by electrophoresis on an agarose gel (data not shown). Reaction mixtures (total volume = 20 µL) contained 3 ng of template DNA, 1X SYBR Green Master mix (4 µL), 500 nM of each forward and reverse primer (2 µL) and of 1X ROX (0.4 µL). QPCR was initiated with a preincubation phase of 10 min at 95^°^C followed by 40 cycles of 95^°^C denaturation for 10 sec and the temperature for primer extension for 10 sec. A final extension at 72^°^C for 10 sec (in small amplicons) or 50 sec (in large amplicons) was performed. The relationship between the threshold cycle and the DNA dilution values was linear with a correlation coefficient R^2^ higher than 0.9 in all cases (data not shown). This method showed linearity over a wide range of template concentrations, therefore it is an accurate technique for DNA damage determination.

#### Lesion rate

In order to calculate the number of lesions induced by cryopreservation in the studied zebrafish genes, a quantitative PCR amplification and fluorescence detection was carried out in a Step 1 Plus system (Applied Biosystems, Spain), according to guidelines provided by the manufacturer. Step 1 Free Software v.2.0 (Applied Biosystems, Spain) was used to calculate crossing point values. Technical and biological triplicates were done. The difference in the threshold cycle (Cts) between long fragments and short fragments (internal normalization control) was determined. The number of lesions was calculated employing the previously published formula by Rothfuss and colleagues [33]. The zebrafish primer nucleotide sequences, annealing temperatures and sizes of amplicons can be found in the supplementary material (Table S2).

$$\text{Lesion}\;\;\text{rate}\;\text{[Lesion}\;\text{per}\;\text{10}\;\text{kb}\;\text{DNA]}\;=\text{(}\;\text{1}-{\text{2}}^{-\text{(}\Delta \text{Ctlong}-\text{ΔCtshort)}}\text{)}\;\text{X}\;\frac{\text{1000}\;\text{[bp]}}{\text{size}\;\text{of}\;\text{long}\;\text{fragment}\;\text{[bp]}}$$

#### Data analysis

DNA lesions were estimated as lesions per 10 kb DNA, including the size of the long amplicon. Results are represented as the means ± SE of the number of lesions per 10 kb of three independent experiments with three replicates for each. Statistical differences in DNA damage were analyzed by one way ANOVA followed by the Student-Newman-Keuls (SNK) method used for comparisons after the performed analysis of variance as a *post hoc* test. All the statistical analyses were conducted using the Statistical Product and Service Solutions (SPSS), IBM, v.20 software.

### Gene expression assay

#### RNA isolation and DNase treatment

RNA was extracted from zebrafish GRs containing PGCs in fresh, cryopreserved and CPAs exposed samples (approximately 75 GRs per replicate) and human sperm (approximately 5 million per replicate) using Trizol reagent (Invitrogen, Madrid) according to the manufacturer’s protocol. RNA quantity and purity were determined using a nanodrop spectrometer (nanodrop 1000, Thermo Scientific, Biocompare, Spain). This protocol also includes a DNase I treatment step. The isolated RNA showed high purity (A_260/280_ > 1.8) and was stored at -80 ^°^C until further use.

#### Reverse transcription

Complementary DNA (cDNA) was obtained from RNA (1µg) using the cDNA synthesis kit (Invitrogen, Madrid), following the manufacturer’s protocol. The cDNA for zebrafish and human samples was stored at -20 ^°^C before analysis by qPCR. In zebrafish GRs containing PGCs and human sperm, reverse transcription (RT-PCR) conditions were 95^°^C for 3min, and 35 cycles of: 95^°^C for 30 sec, 62^°^C for 30 sec and 72^°^C for 30 sec, and a final extension at 72^°^C for 10 min.

In human sperm samples (fresh and cryopreserved), eukaryotic translation initiation factor 2, subunit 1 alpha (e*IF2S1*) with an important role in protein synthesis, homeobox B1 (*HOXB1*) reported as marker of pregnancy success and luteinizing hormone/choriogonadotropin receptor (*LHCGR*) considered as human male quality marker [6], were analyzed.

#### Real time qPCR

In order to measure gene expression in zebrafish GRs containing PGCs (fresh, treated with CPAs and cryopreserved GRs) and in human sperm samples (fresh and cryopreserved), qPCR was performed. The primers for qPCR were designed using Primer Express (Software v2.0, Applied Biosystems) and Primer Select (Software v 10.1 DNA Star, Lasergene Core Suit). The primer nucleotide sequences and annealing temperature from human sperm and zebrafish genes can be found in Supplementary material (Table S3).

The qPCR conditions were optimized for the different primers to achieve similar amplification efficiencies to compare different amplicons. Product specificity was tested by melting curves and product size was visualized by electrophoresis on agarose gel (data not shown).

Reaction mixtures (total volume = 20 µL) contained 2 µL of cDNA, 1X SYBR Green Master mix (Applied biosystems, Madrid) (10 µL) and 500 nM each forward and reverse primer (2 µL).

QPCR was initiated with a preincubation phase of 10 min at 95^°^C followed by 40 cycles of 95^°^C denaturation for 10 sec and the temperature for primer extension for 60 sec. The relationship between the threshold cycle and the dilution values of DNA was linear with a correlation coefficient R^2^ higher than 0.9 in all cases (data not shown). Each experiment was performed three times and three technical replicates were done per sample.

Expression levels for each zebrafish gene relative to *actb2* and for each human gene relative to human *ACTB*, were calculated for all zebrafish and human samples using the delta-delta-Ct (2^−ΔΔCt^) method, which is an algorithm to analyze the relative changes in gene expression. It requires the assignment of one housekeeping gene, which is assumed to be uniformly and constantly expressed in all samples [38]. The *actb2* stability after cryopreservation was validated by our group using qPCR (Supplementary Figure S1).

#### Data analysis

Data were analyzed using SPSS V.16 (IBM, USA) and Microsoft Excel. All results were expressed as the means ± SE of the 2^−ΔΔCt^ method of three independent experiments with three replicates for each. The Student’s t-test (μ=1) was performed to identify changes in gene expression levels after cryopreservation.

### Methylation analysis

#### DNA conversion with bisulphite

Genomic DNA from fresh and cryopreserved zebrafish GRs containing PGCs was bisulphite converted using the EpiTect Bisulphite Kit (Qiagen, Spain).

#### Bisulphite sequencing

Converted DNA was amplified by PCR and nested PCR using primers described by Wu et al. [24] and Lindeman et al. [39]. The sequencing primers and specific annealing temperature are listed in the supplementary material and methods section (Table S3). PCR conditions were 95^°^C for 7 min and 40 cycles of 95^°^C for 1 min, annealing temperature (see Table S3) for 2 min and 72^°^C for 2 min, followed by 10 min at 72^°^C. Size and sequences of PCR amplicons are listed in Supplementary table S4. PCR products were cloned into *E. coli* DH5α TOPO TA cloning and sequenced. The CpG viewer software for DNA methylation analysis was used [40].

## Results

### Cryopreservation induces different levels of DNA damage in zebrafish genes

**Figure 1:**
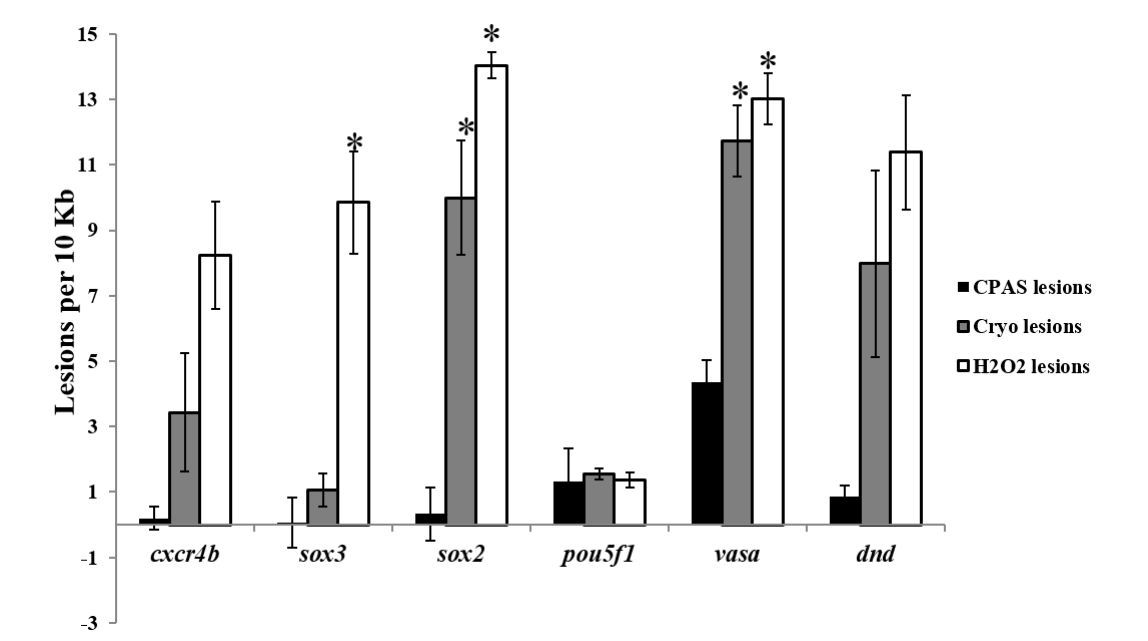
Number of DNA lesions per 10 kb in specific genes. Cryopreservation induces different levels of DNA damage in zebrafish genes. DNA was extracted from fresh, incubated in cryoprotectants (CPAs), cryopreserved (Cryo) and H_2_O_2_ treated (3% for 30 min) (H_2_O_2_) zebrafish genital ridges. The formula employed for lesion rate calculation [33] estimates the number of lesions in different treated samples in comparison with fresh control samples (no lesions). Three independent experiments were done and three replicates were carried out in each of them. Error bars indicate standard error (SE). Results are represented as the mean ± SE. Statistical differences were analyzed by one-way ANOVA followed by SNK as a *post hoc* test. Asterisks represent significant differences (p< 0.05) between treatments for each gene.

As expected, the highest number of lesions was found in peroxide treated zebrafish samples, ranging from 8.24±1.64 lesions in *cxcr4b* to 14.04±0.41 lesions in *sox2*. The number of lesions was surprisingly low in *pou5f1* after peroxide treatment (1.36±0.23 lesions). Cryopreservation significantly increased the number of lesions in 2 of the 6 studied genes (*sox2* and *vasa*). After cryopreservation, the minimum number of lesions was found in *sox3* and *pou5f1* genes (1.05±0.50 and 1.55±0.16 lesions, respectively). The highest values of DNA damage after cryopreservation were observed in *sox2* and *vasa* genes (9.99±1.75 and 11.72±1.08 lesions respectively) (Figure 1).

The results demonstrated that *pou5f1* and *sox3* were less prone to DNA damage induced after the cryopreservation process, *pou5f* being particularly resistant even to peroxide treatment.

### Cryopreservation affects gene expression levels in zebrafish genital ridge and alters transcripts in human sperm

**Figure 2:**
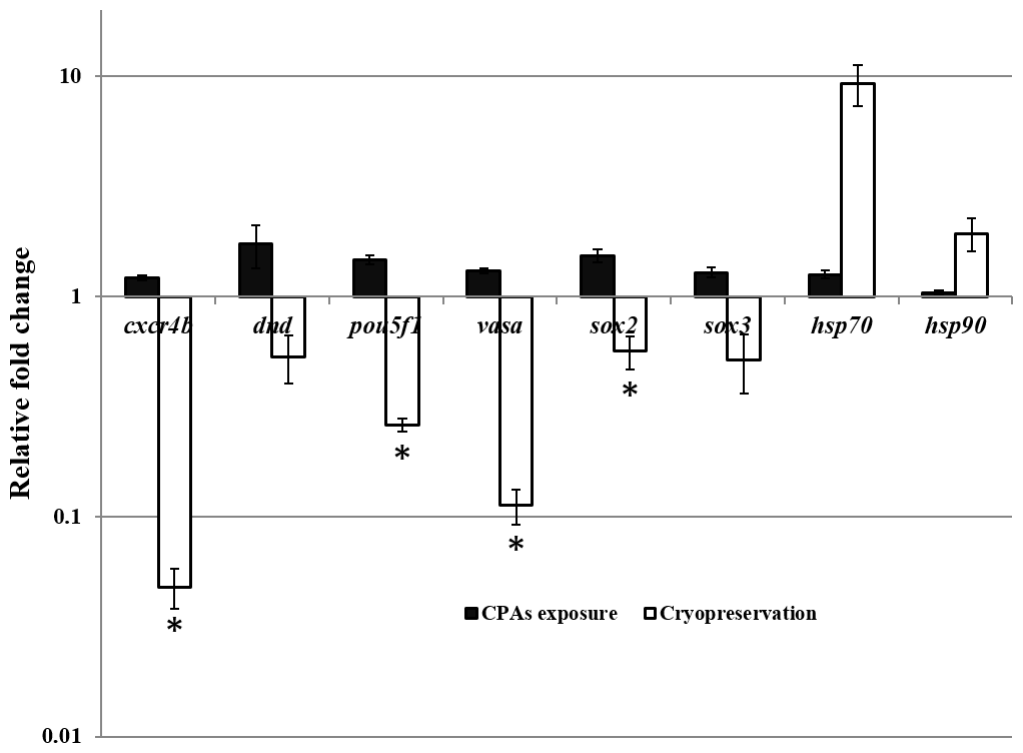
Relative expression of zebrafish genes after CPAs exposure (CPAs) and cryopreservation (Cryo). Cryopreservation affects gene expression levels in zebrafish genital ridges. Analysis was carried out in zebrafish genital ridges (GRs) containing primordial germ cells (PGCs). Expression levels for each gene relative to *actb2* were calculated for all samples using the 2^−ΔΔCt^ method. All results were expressed as the means ± SE of the 2^−ΔΔCt^ method of three independent experiments with three replicates for each. The Student’s t-test (μ=1) was performed to identify changes in gene expression levels after cryopreservation. Asterisks showed significant (p< 0.05) downregulation after cryopreservation (Cryo).

In order to study the effect of cryoprotectant exposure and cryopreservation on zebrafish gene expression, qPCR analysis was performed in zebrafish GRs treated with CPAs and GRs cryopreserved. Expression levels were calculated for all samples using the 2^−ΔΔCt^ method and *actb2* as reference gene. After cryopreservation, all the studied genes (*cxcr4b, dnd, pou5f1, vasa, sox2* and *sox3*) were downregulated. The decrease in transcripts was significant for: *cxcr4b*, *pou5f1, vasa* and *sox2* genes (Figure 2). However, heat shock proteins (*hsp70* and *hsp90*), associated with osmotic and thermal stress [41], were upregulated (Figure 2). When GRs containing PGCs were incubated with CPAs but not subjected to freezing and thawing, no downregulation was observed in the studied genes and no upregulation in the heat shock proteins was found (Figure 2).

**Figure 3:**
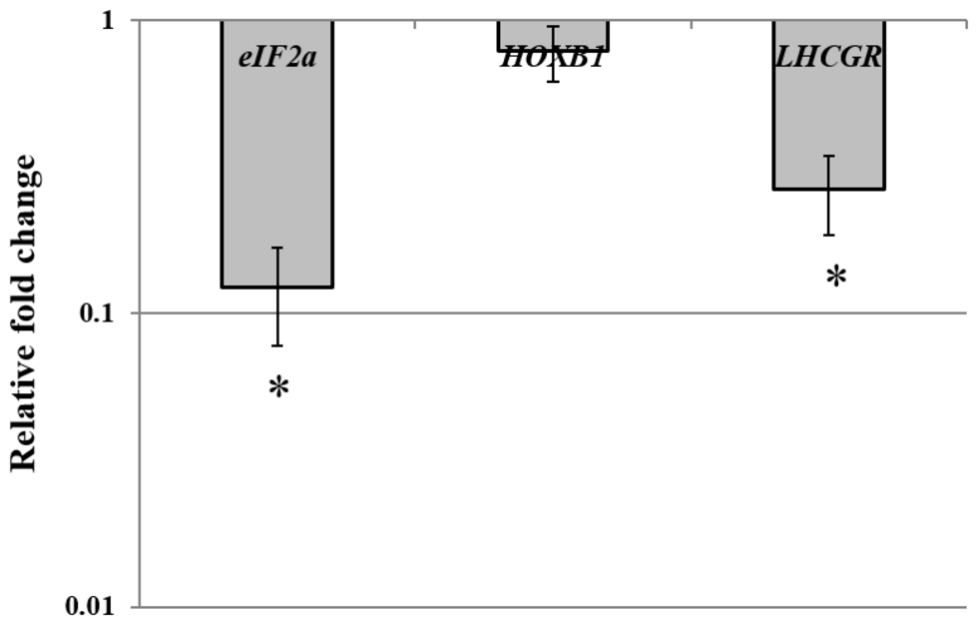
Relative expression of human genes after cryopreservation (Cryo). Cryopreservation affects expression of some transcripts in human sperm. Analysis was carried out in human spermatozoa, transcriptionally inactive cells. Expression levels for each gene relative to *ACTB* were calculated for all samples using the 2^−ΔΔCt^ method. All results were expressed as the means ± SE of the 2^−ΔΔCt^ method of three independent experiments with three replicates for each. The Student’s t-test (μ=1) was performed to identify changes in gene expression levels after cryopreservation. Asterisks showed significant (p< 0.05) downregulation after cryopreservation (Cryo).

In human spermatozoa (transcriptionally inactive cells), qPCR results demonstrated that cryopreservation can affect the presence of some transcripts (*eIF2S1* and *LHCGR*) whereas others could remain intact (*HOXB1* and *ACTB*) (Figure 3).

### Different methylation levels were found after cryopreservation of zebrafish GRs, containing PGCS

**Figure 4:**
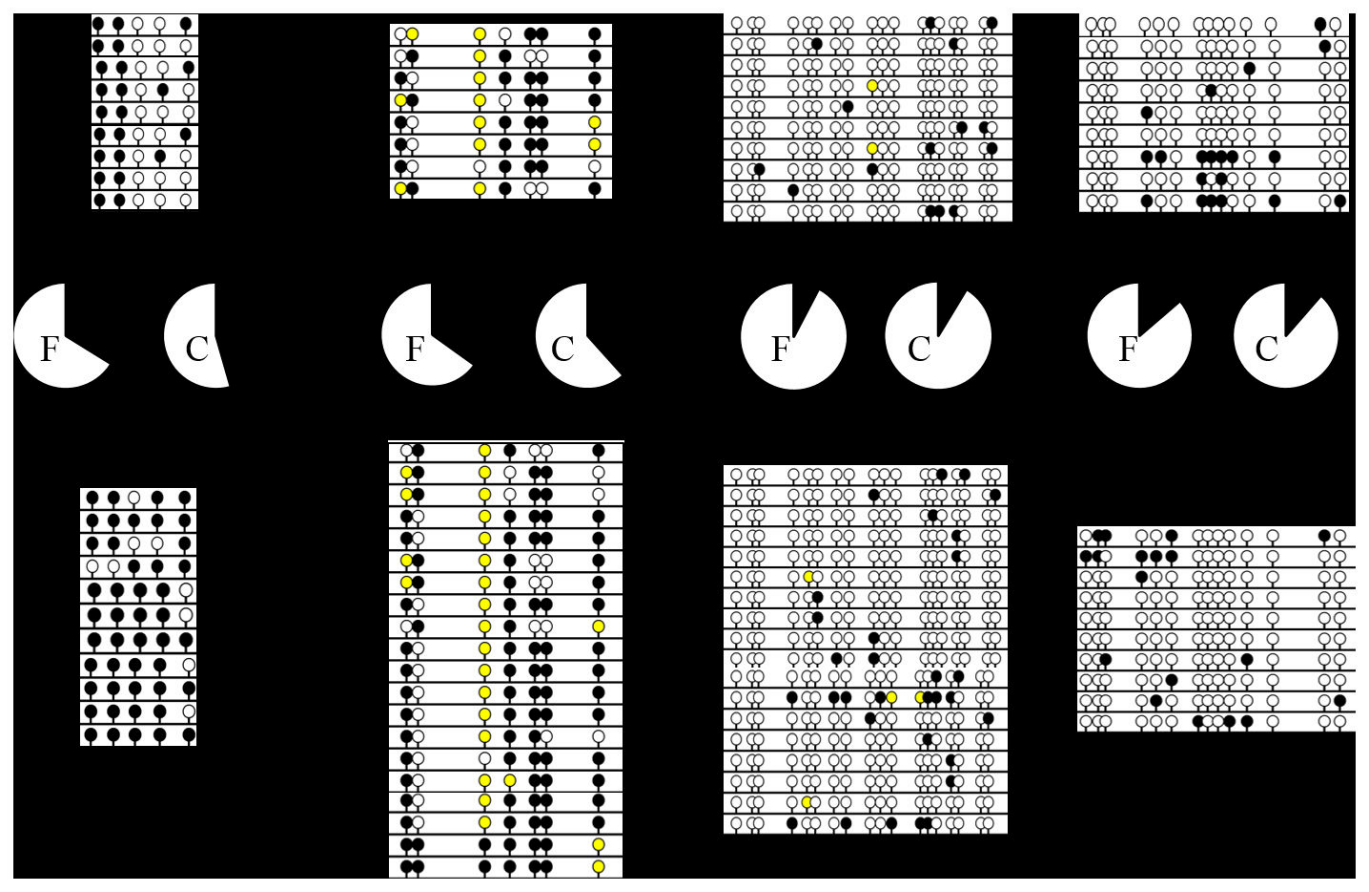
Bisulphite sequencing analysis of CpG methylation in the promoter of *cxcr4b* (A)*, pou5f1* (B)*, sox3*(C) and *vasa* (D) genes in fresh and cryopreserved zebrafish genital ridges (GRs) containing primordial germ cells (PGCs). Different methylation levels were found after cryopreservation of zebrafish GRs. White circles represent methylated CpG and black circles unmethylated islands. The software represented islands with an undetermined methylation pattern with yellow circles. The percentage of methylated CpGs for each promoter was represented.

To study whether differences in zebrafish gene expression after cryopreservation could be associated with a promoter methylation, we assessed the DNA methylation profile using bisulphite genomic sequencing. The highest methylation levels after cryopreservation were found in *vasa* (83.6%) (Figure 4A) and *cxcr4b* (62.14%) (Figure 4B) promoters, in agreement with their significantly repressed state. In contrast, in *pou5f1*, (Figure 4C) which also presented a significant downregulation profile after cryopreservation, no differences in DNA methylation were found in cryopreserved GRs.

## Discussion

Cryopreservation is a technique commonly used for gene banking purposes and is routinely employed in ARTs. Human sperm, oocyte and embryos have been cryopreserved for decades. It is well known that reactive oxygen species (ROS) can be produced during the process and these free radicals can damage DNA [42–44]. Possible damage produced in the DNA is fragmentation. In fact, this is almost the only parameter related to DNA status assessed after cryopreservation in routine protocols. However, it is well known that many other types of DNA damage can occur. This damage could be particularly serious if it affects genes that are important in early embryo development. Recent technology has enabled the quantification of DNA damage in specific genome regions [33]. Our group has employed this technology for the first time in order to quantify the number of lesions produced by cryopreservation in specific nuclear and mitochondrial genome regions [45].

In this candidate gene approach, we used the technique to assess the effect of cryopreservation on the key zebrafish genes being studied. Our results showed that *pou5f1* and *sox3* were less prone to DNA damage induced by the cryopreservation process. In contrast, significant differences in DNA damage after cryopreservation were observed in *sox2* and *vasa* genes (Figure 1). Differences in susceptibility between genes were clearly detected and could be at least partially attributed to the different position that the specific DNA regions have within the nuclei (nuclear territories). It is known that regions closer to the nuclear envelope may be more susceptible to damage [46]. Different panels of genes could be studied by this technique depending on the cell type cryopreserved. In our case, *vasa* is a crucial gene for PGCs since it is involved in germ cell lineage development [28]. In zebrafish, *vasa* transcripts become targeted to the cleavage planes of early embryos and subsequently incorporated into the PGCs [34]. Therefore damage in this gene could have undesirable effects should these cells be used for cryobanking. This is particularly interesting considering that this cryopreservation protocol did not decrease cell survival, DNA integrity or cell functionality in comparison with the fresh control [26]. Similarly, there are several genes considered crucial for early embryo development in humans (*pou5f1, nanog, sox2, klf4, etc*) [47]. Guaranteeing the absence of damage after human gamete and/or embryo cryopreservation in those genes could be particularly important.

The reduction of some transcripts or even the elimination of some of them as a consequence of cryopreservation has also been reported by different groups [6]. In our study, we observed a downregulation of all the zebrafish studied genes, *cxcr4b, dnd, pou5f1, vasa, sox2* and *sox3* (Figure 2). This downregulation was significant in *cxcr4b*, *pou5f1*, *vasa* and *sox2* (Figure 2). However, taking into account that, GRs containing PGCs were not subjected to further culture after cryopreservation we questioned whether there was enough time during the process for transcriptional regulation. We assessed the expression of two genes involved in thermal and osmotic shock (*hsp* 70 and 90) [41] after cryopreservation we observed a clear upregulation of those transcripts, suggesting that transcriptional regulation is possible. However, a modification in gene expression could be caused by CPA incubation rather than by freezing/thawing itself. We evaluated gene expression in zebrafish GRs containing PGCs exposed to cryoprotectants but not subjected to cryopreservation, and we observed neither downregulation of the studied genes nor upregulation of the heat shock proteins (Figure 2). All these data suggested that changes in gene expression could occur mainly during freezing/thawing. At this point we questioned whether the decrease in transcripts could always be explained by a repression mechanism of transcription. Methylation of cytosine residues in DNA is considered to be one of the major epigenetic mechanisms controlling gene expression and imprinting [48]. Recent studies have shown a negative correlation of sperm DNA fragmentation and DNA methylation. Tunc and Tremellen [49] suggested that oxidative stress-related damage to sperm DNA impedes the process of methylation, while antioxidant supplementation appears to have the potential to reduce DNA damage and normalize sperm DNA methylation. O^6^-methylguanine and 8-hydroxyl-2´-deoxyguanosine (8-OH-dG) were both reported to interfere with DNA ability to act as a substrate for DNA methyltransferases [50]. Since our cryopreservation protocol guarantees the absence of DNA fragmentation [26], DNA methylation analysis can be carried out avoiding the above mentioned effect. We found that, different methylation levels were found after cryopreservation among the studied genes, however, the epigenetic status (promoter methylation) did not always correlate with gene expression. Only in some genes (*vasa* and *cxcr4b*), did cryopreservation produce an increase in methylation that could be correlated with the downregulation observed (Figure 2 and Figure 4A and 4B). But, what about the other transcripts? Is a decrease in transcripts possible without a decrease in transcription? In order to answer these questions we used transcriptionally inactive cells, human spermatozoa. To study the effect of cryopreservation on human spermatozoa at the level of transcripts, it is extremely important to take into account the importance of some of their transcripts in fertility and early embryo development. Transcriptional activity has never been demonstrated in spermatozoa [51]. Therefore it can be assumed that mRNA molecules present in spermatozoa are a remnant of the transcripts from spermatogenesis [52] and any decrease in transcripts observed in these cells after cryopreservation could not be explained by epigenetic mechanism. Our results demonstrated that human *HOXB1*, reported to be a marker of pregnancy success, remains stable whereas e*IF2S1*, with an important role in protein synthesis, and *LHCGR*, considered to be a human male quality marker [6], decrease after cryopreservation (Figure 3). This experiment allowed us to conclude that in some cases, modifications in transcripts caused by cryopreservation should be explained by other mechanisms that do not involve methylation modifications. As a possible explanation for this observation, we can hypothesize, that cryopreservation affects mRNA stability making some of these molecules more susceptible to degradation. Recent studies showed that the regulation of some mRNAs involves not only translation inhibition, but also changes in the polyA tail length, which affect both translation efficiency and mRNA stability. It is known that some proteins repress the expression of specific maternal mRNAs. mRNAs associated with CUP (an eIF4E-binding protein) could be either fully degraded or stored in a repressed deadenylated form [52–55]. Similar mechanisms could be acting on paternal mRNAs, since some transcripts should be kept stable to be transferred to the oocyte. It is a fact that cryopreservation can alter the nucleoprotein structure in sperm [56] and, in a similar way, cryopreservation may affect mRNA stability by altering their association with certain proteins.

We can conclude that optimized cryopreservation protocols could have a clear effect, not only causing DNA lesions but also reducing crucial transcripts, in some cases producing hypermethylation. If these modifications occur in the paternal contribution (genes or transcripts) they could perhaps be repaired (in the case of DNA damage by error-free homologous recombination pathway or by non-homologous end-joining) or overcome (in the case of transcript alterations by being compensated by maternal transcripts or after zygotic transcription) in the embryo, however this should be carefully studied in order to ensure the total efficiency and safety of the technique.

## Supporting Information

Table S1 Summary of gene abbreviations, gene complete names and gene functions in zebrafish primordial germ cells (PGCs) and in human sperm. (TIF)

Table S2 List of primers for DNA damage. Sequences start from 5´ to 3´. Annealing temperature and amplicon size is specified for each pair of primers. (TIF)

Table S3 List of primers for gene expression and for bisulphite sequencing. Sequences start from 5´ to 3´. Annealing temperature and the species are specified for each pair of primers. (TIF)

Table S4 Sequences, amplicon size and number of GpCs islands of the analyzed genes from Figure 4. Underlined CG corresponds with the analyzed CpG islands in the zebrafish genes studied before and after cryopreservation (*cxcr4b* -240 bp-, *pou5f1* -255 bp-, sox 3-274 bp- and *vasa* -156 bp-).
(TIF)

Figure S1 *actb2* Ct values means in fresh and cryopreserved genital ridges (GRs) from 26 somite zebrafish embryos. Ct values mean ± SE of three independent experiments with three replicates for each. Ct values remained unaffected by cryopreservation.
(TIF)
