# Correction: Cryopreservation Causes Genetic and Epigenetic Changes in Zebrafish Genital Ridges

**DOI:** 10.1371/annotation/3265139d-64c7-4c4c-83d3-1e139031e7df

**Published:** 2014-01-10

**Authors:** Marta F. Riesco, Vanesa Robles

Figure 4 has been updated for improved readability. Please see the correct Figure 4 here: 

**Figure:**
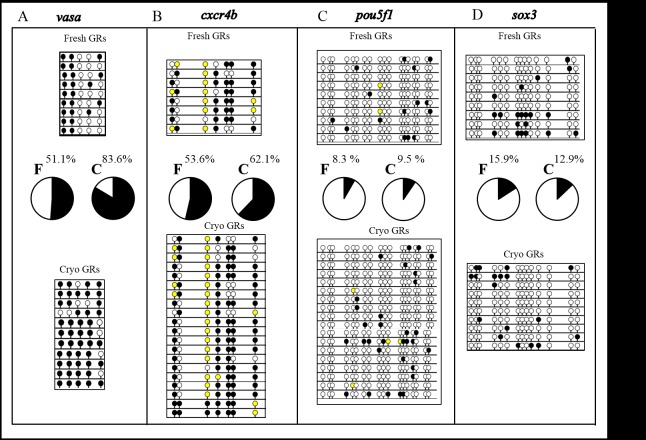


The legend for Figure 4 is:

**Bisulphite sequencing analysis of CpG methylation in the promoter of *vasa* (A), *cxcr4b* (B), *pouf1* (C) and *sox3* (D) genes in fresh and cryopreserved zebrafish genital ridges (GRs) containing primordial germ cells (PGCs).** Different methylation levels were found after cryopreservation of zebrafish GRs. For two of the four promoters the methylation level increased following cryopreservation: for *vasa* there was a 32.5% increase (p<0.05), and for *cxcr4b* a 8.5% increase (p<0.05), whereas for *pou5f1* and *sox* 3 the changes were not statistically significant. White circles represent un-methylated CpGs, black circles methylated ones, whereas yellow circles those with an undetermined methylation pattern. The percentage of methylated CpGs for each promoter is shown above the circles depicting the overall proportion of methylation.

